# Supplementary material for: Hybrid Models and Biological Model Reduction with PyDSTool
Source: PLoS Comput Biol. 2012 Aug 9;8(8):e1002628. doi: 10.1371/journal.pcbi.1002628 (PMC3415397; doi:10.1371/journal.pcbi.1002628)
Supplement: Text S4 — Complete source code for the PyDSTool package (version 0.88.120504). Includes API documentation and help files linking to web pages. This file is identical to the current public release on Sourceforge.net. (ZIP) [file pcbi.1002628.s004.zip › PyDSTool/html/PyDSTool.PyCont.Plotting-module.html]

xml version="1.0" encoding="ascii"?


PyDSTool.PyCont.Plotting


| Home | Trees | Indices | Help | PyDSTool |
| --- | --- | --- | --- | --- |

Package PyDSTool ::
Package PyCont ::
Module Plotting

[hide private]

[frames] | no frames]

# Module Plotting

source code

Plotting class and function

Drew LaMar, May 2006


|  |  |
| --- | --- |
| Classes | [hide private] |

pargs

KeyEvent  
Used in 'highlight' method of plot\_cycles.


|  |  |
| --- | --- |
| Functions | [hide private] |

|  |  |
| --- | --- |
| initializeDisplay(plot, figure=None, axes=None)  If figure = 'new', then it will create a new figure with name fig# If figure is an integer, that figure # will be selected | source code |


|  |  |
| --- | --- |
| Variables | [hide private] |

all\_point\_types = `['P', 'RG', 'LP', 'BP', 'H', 'BT', 'ZH', 'CP...`

all\_curve\_types = `['EP', 'LP', 'H', 'FP', 'LC']`

\_classes = `['pargs', 'KeyEvent']`

\_functions = `['initializeDisplay']`


|  |  |
| --- | --- |
| Variables Details | [hide private] |

### all\_point\_types

Value:
:   ```
    ['P',
     'RG',
     'LP',
     'BP',
     'H',
     'BT',
     'ZH',
     'CP',
    ...
    ```

  


| Home | Trees | Indices | Help | PyDSTool |
| --- | --- | --- | --- | --- |

|  |  |
| --- | --- |
| Generated by Epydoc 3.0.1 on Fri May 4 15:24:03 2012 | http://epydoc.sourceforge.net |
